# Mapping cellular processes that determine delivery of plasmid DNA to the nucleus: application in Chinese hamster ovary and human embryonic kidney cells to enhance protein production

**DOI:** 10.3389/fbioe.2025.1466671

**Published:** 2025-03-21

**Authors:** James D. Budge

**Affiliations:** School of Natural Sciences, University of Kent, Canterbury, United Kingdom

**Keywords:** transfection, DNA delivery, nuclear import, mammalian cell culture, Chinese hamster ovary (CHO) cells, cell cycle, recombinant protein production, human embryonic kidney (HEK) cells

## Abstract

Delivery of DNA into nucleated eukaryotic cells is known as transfection and has been essential in establishing technologies such as recombinant protein production and gene therapy. Considerable research efforts have led to development of a variety of transfection methods for a multitude of applications and cell types. Many methods are efficient in delivering DNA across the plasma membrane but few focus on subsequent delivery into the nucleus, a necessary step in expression of a recombinant transgene, and the cellular processes governing nuclear import of DNA during transfection have proved elusive. Herein, live confocal microscopy was used to track plasmid DNA during transfection of Chinese hamster ovary (CHO) and human embryonic kidney (HEK) cells to map key cellular processes central to nuclear import of DNA showing that there is a strong relationship between events of cell division, promotion of DNA dispersal from endosomes and subsequent nuclear import leading to gene expression. Furthermore, cationic lipid-mediated transfection is more dependent on events of the cell cycle than electroporation to deliver DNA into the nucleus. These findings have informed the design of a method where both CHO and HEK cells are synchronised at G2 phase of the cell cycle followed by timely release enabling cell cycle progression to maximise the frequency of division events immediately after transfection. This led to a 1.2–1.5 fold increase in transfection efficiency for polyethylenimine (PEI) mediated and electroporation transfection respectively. This process enhanced production yields of a monoclonal antibody 4.5 fold in HEK and 18 fold in CHO cells in the first 24 h post transfection. Overall, this study elucidated key cellular processes fundamental to transfection of CHO and HEK cells providing knowledge which can be applied to DNA delivery technologies in a plethora of fields.

## 1 Introduction

Transfection is the method of introducing foreign nucleic acids into a cell to alter its properties. The process has a vast array of applications including, but not limited to, large scale protein production, stable cell line generation, viral particle production, gene silencing, stem cell differentiation, gene therapy delivery and studying genes or proteins in a variety of cell types (Chong et al., 2021). Chinese hamster ovary (CHO) cells are the most commonly utilised host for production of complex, multi-domain recombinant biotherapeutics such as monoclonal antibodies as they are robust and have the capacity to produce recombinant molecules at high yields with the required post-translational modifications such as glycosylation (Walsh and Walsh, 2022). Human embryonic kidney (HEK) cells are also employed for the production of monoclonal antibodies, albeit less frequently than CHO, but are the most commonly utilised production host for viral vectors such as lentivirus and adeno-associated virus (AAV). These viral systems have gained traction for delivery of gene therapies and several products have attained FDA approval and the number of these therapies entering clinical trials has increased substantially over recent years. AAV production requires expression of replication (*Rep*) and capsid (*Cap*) genes and packaging of an AAV cis-plasmid comprising the gene of interest and necessary genetic elements to induce packaging and expression. The most commonly employed production technique for AAVs involves triple transfection of three plasmids harbouring the aforementioned components and achieving optimal yields is dependent on efficient transfection of these DNA constructs into HEK hosts. Moreover, recombinant gene expression in both CHO and HEK cells is dependent on efficient transfection methods which facilitate the delivery of DNA expression vectors (usually plasmid DNA) into the cell which can be achieved using a plethora of different techniques generally categorised into viral, chemical and physical/mechanical methods (Kim and Eberwine, 2010). Whilst many of these approaches are extremely efficient in delivering DNA across the plasma membrane into the cytoplasm, very few focus on subsequent delivery into the nucleus which is essential since the cellular machinery for transcription of DNA into RNA is located in the nucleus. Since transcription is an early and vital step in expression of recombinant protein production, migration of DNA into the nucleus is a potential bottleneck in generating a protein of interest at the desired yield in nucleated eukaryotic cells such as CHO cells.

**FIGURE 1:**
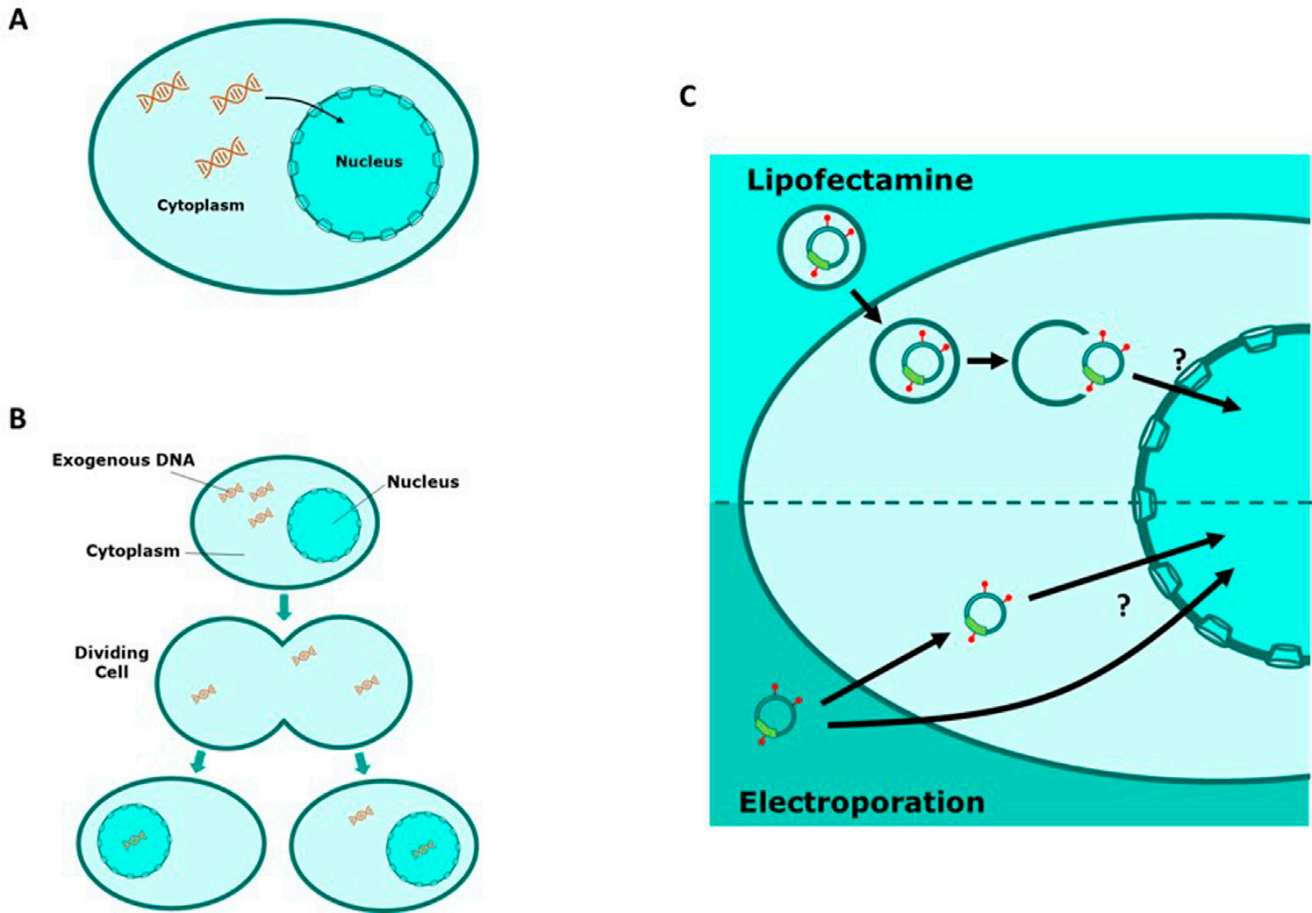
Schematic representations of exogenous DNA migrating to the cell nucleus. **(A)** “Active” nuclear import involves transport of DNA from the cytoplasm into the nucleus via the nuclear pore complex (NPC). **(B)** “Cell cycle dependent” nuclear import relies on degradation of the nuclear membrane during cell division events. DNA is then passively encompassed in the nucleus upon reformation of the nuclear membrane in daughter cells. **(C)** A schematic of cellular events involved in nuclear import of rhodamine tagged plasmid DNA using different transfection methods.

Despite the wide range of applications of transfection, the process by which DNA is imported into the nucleus is not well understood although two major mechanisms have been proposed (Figure 1). Firstly, “active” import theorises that exogenous DNA migrates into the intact nucleus via the nuclear pore complex (NPC) (Dean et al., 2005). Under normal physiological conditions many small molecules can diffuse through the nuclear membrane but it has also been shown that larger molecules can migrate via the NPC in a Ran-GTP dependent manner (Budge et al., 2021; Dean, 1997; Lam and Dean, 2010; Munkonge et al., 2009; Pemberton and Paschal, 2005). Several studies have shown that non-dividing cultured mammalian cells can express a transfected transgene of interest which suggests that DNA is able to migrate into an intact nucleus which is consistent with the theory of active DNA import (Akita et al., 2015; Badding et al., 2012; Bai et al., 2017; Dowty et al., 1995; Fasbender et al., 1997; Ludtke et al., 2002; Zou et al., 2010; Sylvers et al., 2023). Alternatively, cell cycle dependent import theorises that migration of exogenous DNA into the nucleus is heavily reliant on events of mitosis during the cell cycle (Bai et al., 2017). During metaphase of mitosis the nuclear membrane is temporarily degraded whilst a new nuclear membrane assembles in each of the daughter cells during telophase. A fraction of transfected DNA that was initially present in the cellular matrix of the parent cell is spontaneously enveloped into the newly formed nuclei of the daughter cells. Several studies have shown that manipulation of the cell cycle can be effective in improving transfection efficiencies in mammalian cells which supports the theory of cell cycle dependent nuclear import (Brunner et al., 2000; Cervia et al., 2018; Golzio et al., 2002; Grosjean et al., 2002; Sylvers et al., 2023).

Cationic polymer and lipid mediated transfection agents such as polyethyleneimine (PEI) and lipofectamine respectively work by formation of a polyplex between polymers or lipids and DNA prior to adding to cells which facilitates DNA delivery via endocytosis. Once in the cytoplasm, the exogenous DNA must be released from the complex in order to migrate into the nucleus; the cellular mechanism behind this process is poorly defined (Haraguchi et al., 2022). *Bus et al.* identify endosomal escape as a potential bottleneck of efficient non-viral gene delivery and describe the three main theories behind endosomal escape which include 1) the “proton sponge” effect 2) polyplex-mediated escape and 3) free polymer mediated escape; despite which, the exact mechanism remains unclear (Bus et al., 2018). On-the-other-hand, electroporation is able to deliver DNA into cells by delivering a short electrical pulse to a DNA and cell mix. Since DNA is negatively charged, the pulse ensures that DNA migrates into cells as a result of the electrical pulse simultaneously perforating the plasma membrane. In this method, exogenous DNA is not in any complex and thus does not have to escape from such a complex and is free to enter the nucleus (Chong et al., 2021; Shi et al., 2018). Similarly to lipid mediated delivery, the mechanism of migration of this naked DNA into the nucleus is poorly defined. It is noted that naked DNA in the cytoplasm is more prone to degradation than complexed DNA (Vandenbroucke et al., 2007). To address this, researchers have developed electroporation techniques with multiple pulses of defined timings and voltages in order to increase the abundance of DNA reaching the nucleus; a process known as nucleofection (Stroh et al., 2010).

In this study, live confocal microscopy has been used to track exogenous plasmid DNA during lipid, polyethylenimine and electroporation mediated transfection approaches to help define the cellular mechanisms involved in transfection and, in particular, nuclear import of exogenous DNA. Results obtained show that successful transfection is dependent on events of the cell cycle in the vast majority of occurrences in lipid and PEI mediated transfected populations as well as electroporated populations. This information was then used to design and implement strategies centred around cell cycle manipulation to improve existing transfection processes. Overall, the study presented herein further defines key cellular processes involved in transfection and nuclear import of exogenous DNA in eukaryotic nucleated cells.

## 2 Methods

### 2.1 Rhodamine tagging of plasmid DNA

The pcDNA5 FRT vector (Invitrogen) containing the *eGFP* gene in the multiple cloning site was the chosen vector for all transfections throughout this investigation as it is a reliable mammalian expression vector which lacks a promoter for expression of the selection marker which was not required for this study. For confocal microscopy based experiments, plasmid DNA was covalently tagged with Rhodamine using the commercially available LabelIT™ nucleic acid labelling kit (TM-Rhodamine MIR 4125; Mirus Bio) following the manufacturer’s instructions where a 0.1:1 (v:w) Label IT™ reagent was used to tag DNA.

### 2.2 Cell culture

#### 2.2.1 CHO cell culture maintenance

**FIGURE 2:**
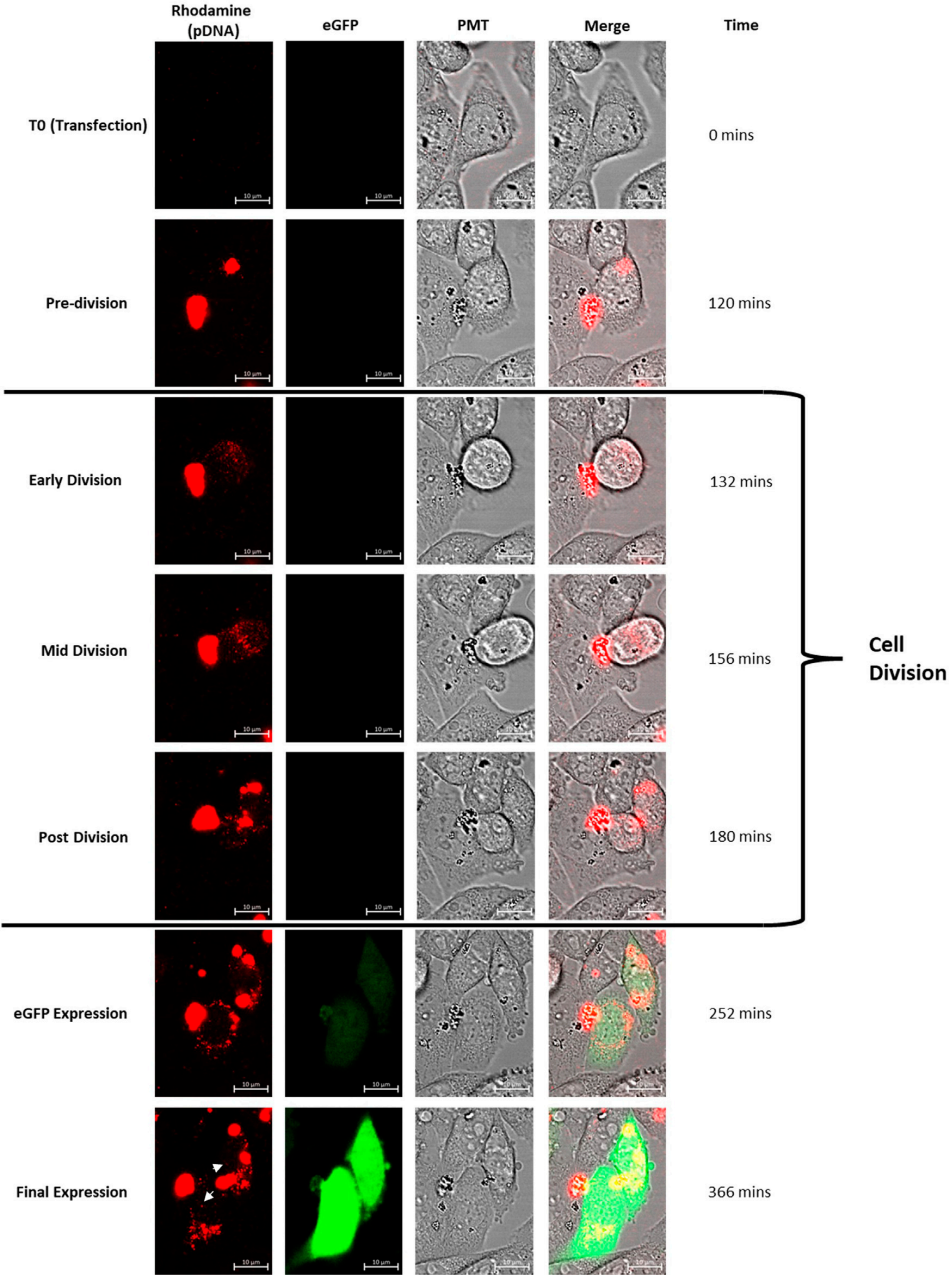
Confocal microscopy images of CHO cells which have been transfected using lipofectamine. Rhodamine has been used to tag the plasmid DNA transfected and the construct bears DNA elements conducive to expression of eGFP. The set of images follow a particular cell (shown using the PMT channel) which divides through the observed time course. Lipofectamine/plasmid DNA mix was added at T0 and the tracked cell clearly contains a concentrated abundance of DNA (as shown in the rhodamine channel) during the pre-division phase. The cell division event initiates with the cell detaching from the culture vessel surface (as shown clearly in the PMT channel) and the tagged DNA is simultaneously dispersed throughout the cell during the “Early Division” stage. The cell then divides and the resulting daughter cells contain plasmid DNA which is interspersed throughout the cell including a small proportion in the nucleus. Both daughter cells then begin to express eGFP (the eGFP expression cassette is present on the tagged plasmid DNA) after the cell division event. This set of images exemplifies the set of events seen in all cells which eventually express the eGFP reporter gene. White arrows indicate punctate spots of rhodamine tagged pDNA evident in the nucleus of the daughter cells at ‘final expression’ image. Multimedia component 1 shows a timelapse video of these events.

**FIGURE 3:**
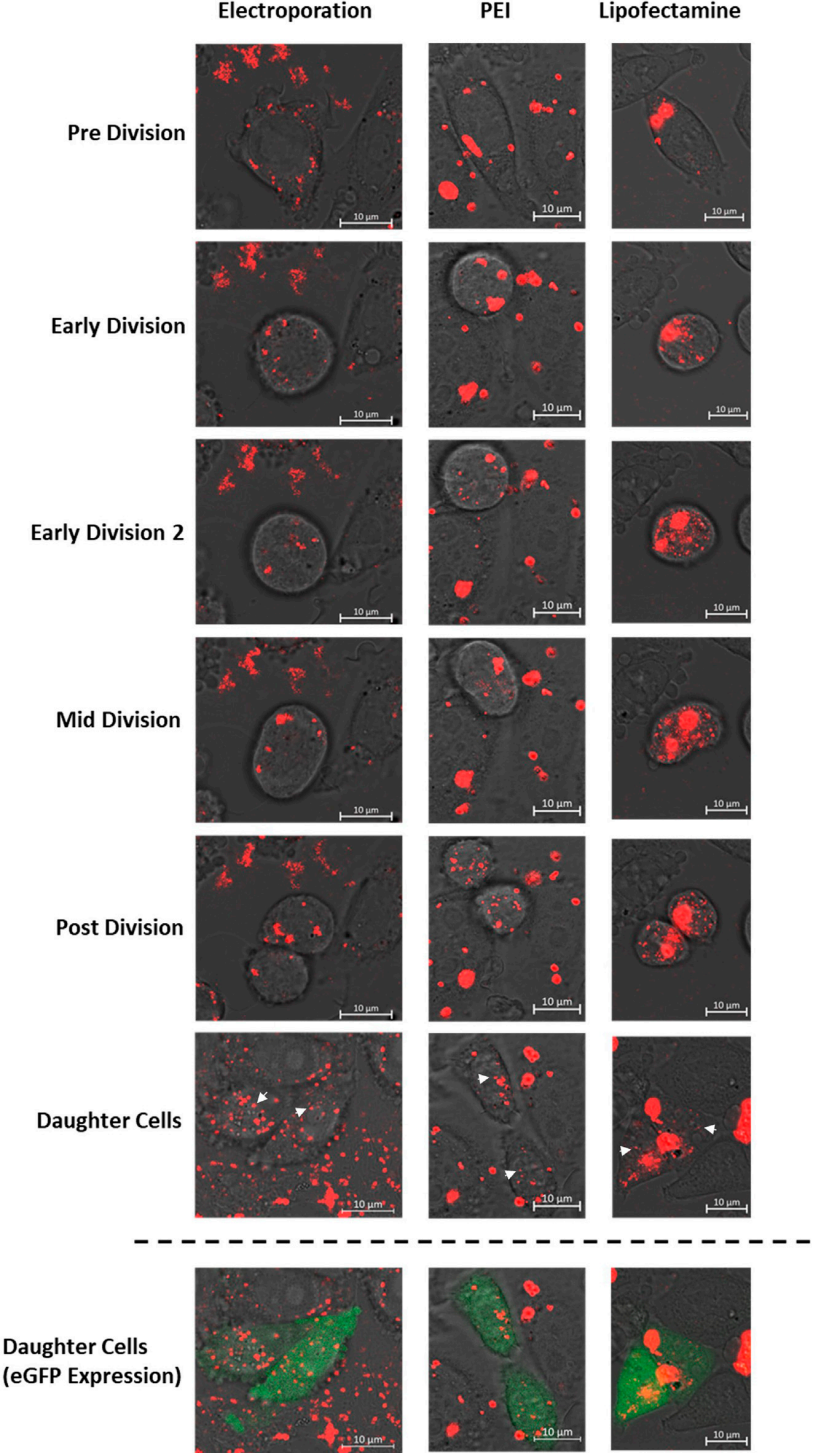
Confocal microscopy images of CHO cells which have been transfected using either electroporation, PEI or Lipofectamine 2000. Transfections were carried out using a plasmid encoding the *eGFP* gene which was labelled with rhodamine prior to transfection. All images show the “rhodamine” and PMT microscope channels which have been merged where the final row of images also shows fluorescence present due to expression of eGFP (green). These data highlight key cellular mechanisms which occurred during mitosis and led to successful expression of the eGFP transgene including 1) endosomal escape of DNA during early division events in PEI and lipofectamine mediated delivery; which is not a feature of electroporation events and 2) successful delivery of plasmid DNA into the nucleus of both daughter cells post cell division. White arrows indicate punctate spots of rhodamine tagged pDNA evident in the nucleus of the daughter cells.

**FIGURE 4:**
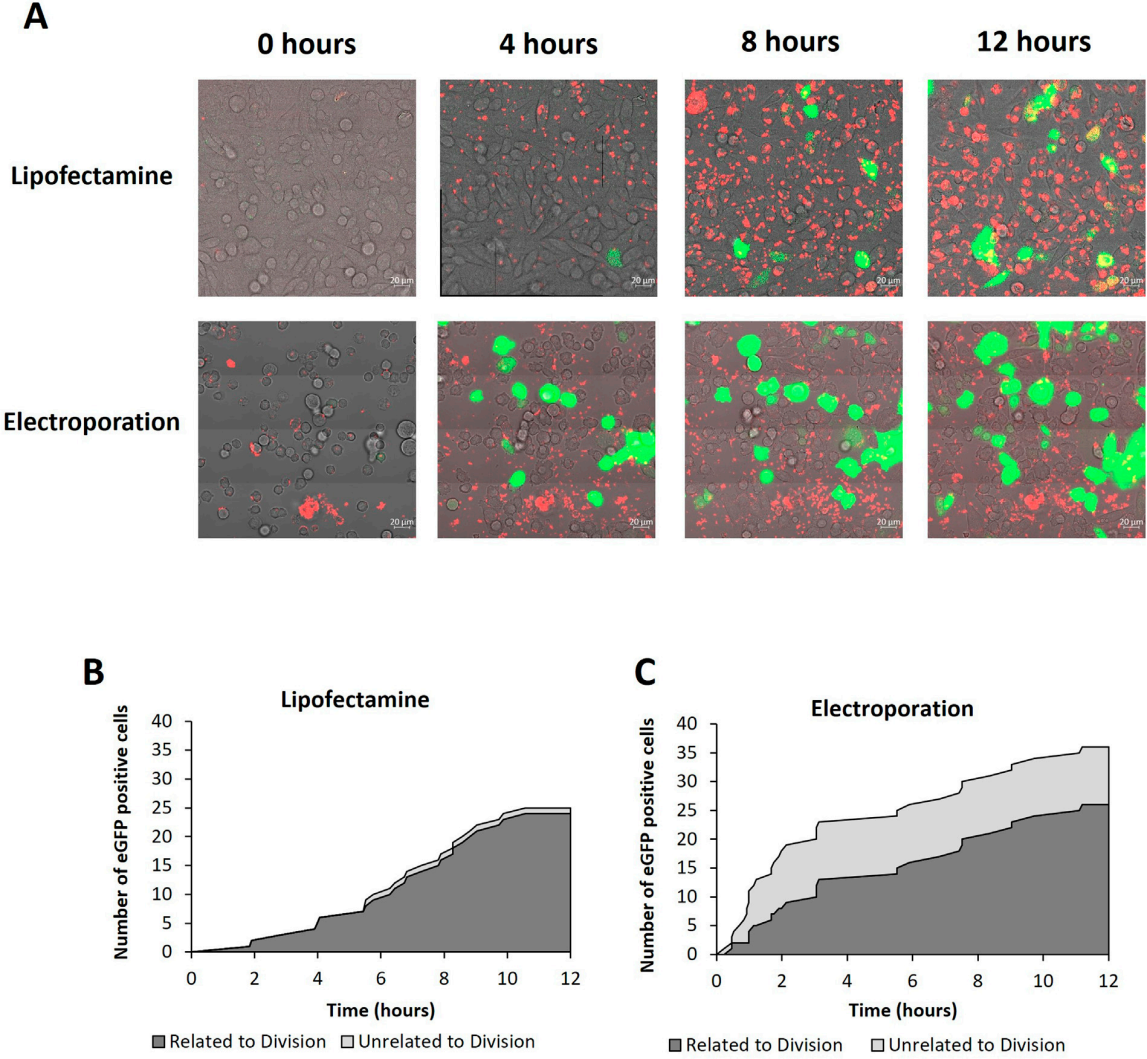
Confocal microscopy analysis of cells transfected with lipofectamine 2000 or electroporation. **(A)** Confocal microscopy images of CHO cells every 4 h of a 12 h time course post transfection using either lipofecatamine 2000 or electroporation as described in methods. Images are a merge of PMT (light microscope), rhodamine labelled plasmid DNA (red) and eGFP (green). Multimedia components show timelapse videos of cells transfected with lipofectamine 2000 and electroporation (Supplementary Video S1). Figures **(B, C)** show analysis of the time course post transfection using lipofectamine 2000 or electroporation, as analysed via confocal microscopy, respectively. The cumulative number of eGFP positive cells are shown and were determined by the images obtained during the time course. Images were collected every 4.6 min and the first evidence of eGFP expression is shown in the graphs. Furthermore, cells which express eGFP have been categorised into expression events which are related to division (i.e., where cells begin the division process prior to expression) or unrelated to division (i.e., where no division event was observed prior to eGFP expression) shown using dark grey and light grey areas respectively.

Both the Flp-In™ CHO cell line (R75807, Invitrogen) and CHO-S cell line (A1155701, Gibco™) were used throughout the study as outlined. Flp-In™ CHO cells were used for confocal microscopy studies (Figures 2–4; Supplementary Figure 1, multimedia component 1, and Supplementary Video S1) as these were able to adhere to confocal dishes and coverslips utilised during these experiments. They were cultured in 10 mL Ham’s F12 Nutrient Mix medium (Invitrogen) supplemented with 10% (v/v) fetal bovine serum (FBS) in T25 flasks at 37°C in a 5% CO_2_ atmosphere. Routine passaging was carried out every 3–4 days and 0.1% Trypsin EDTA was used to detach cells from the surface during passaging. Gibco CHO-S cells were maintained in 20 mL cultures in CD-CHO medium at 37°C in a 5% CO_2_ atmosphere with shaking at 125 rpm (2.5 cm orbit). Cultures were routinely passaged every 3–4 days and seeded at 0.2 × 10^6^ viable cells/mL in 125 mL Erlenmeyer flasks (Corning^®^).

#### 2.2.2 HEK cell culture maintenance

HEK Freestyle™ 293-F cell lines (Invitrogen™, R790-07) were cultured in FreeStyle™ 293 Expression medium maintained in 20 mL culture volumes at 37°C shaking at 125 rpm (2.5 cm orbit) in a 5% CO_2_ atmosphere. They were routinely passaged every 3–4 days seeded at 0.3 × 10^6^ viable cells/mL in 125 mL Erlenmeyer flasks (Corning^®^).

#### 2.2.3 Cell cycle manipulation

Known cell cycle inhibitors were added to cultures to arrest cells in particular phases of the cell cycle. Roscovitine (CAS 186692–46–6, Santa Cruz Biotechnology) was added to a final concentration of 20 µM to prevent cell cycle progression beyond G1 and G2 phases, colcemid (10295892001, Sigma) was supplemented in culture to a final concentration of 0.1 μg/mL to arrest cells in metaphase and the Cdk1 inhibitor RO-3306 (217721, Sigma) was used at a final concentration of 9 µM to arrest cells in G2 phase. When carrying out experimentation involving the cell cycle inhibitors described above, cells were incubated in the presence of the inhibitor overnight (18 h) before utilising. Roscovitine and colcemid stock solutions were diluted in DMSO such that 0.4 µL were added to cell culture medium to achieve the stated concentrations. Controls were also cultured in the same concentration of DMSO appropriately.

#### 2.2.4 Electroporation

A GenePulser Xcell electroporator (Bio-Rad) was used to electroporate CHO and HEK cells where 1 × 10^7^ cells in 0.7 mL medium (either CD-CHO for Gibco CHO-S cells, Ham’s F12 Nutrient Mix for CHO Flp-In cells or FreeStyle™ 293 Expression medium for HEK FreeStyle™ 293-F cells) was combined with 20 µg plasmid DNA diluted in 0.1 mL TE buffer in an electroporation cuvette with a 4 mm electrode gap (BioRad). It has been suggested that water is a more suitable solution for dilution of DNA during electroporation protocols as TE buffer contains ions which can affect the electric field as well as promote internalisation of EDTA also present in the buffer (van den Hoff et al., 1995). Conversely, TE buffer has clear benefits in DNA storage and is therefore frequently utilised in established transfection protocols (Budge et al., 2019). Flp-In CHO cells were detached from the surface using 0.1% trypsin EDTA, resuspended in 10 mL medium and centrifuged at 200 *g* for 5 min before washing with PBS twice to remove any traces of trypsin which can have a detrimental effect on transfection processes. Electroporation was carried out using the exponential protocol using 900 µF resistance and either 250 or 300 V for different experiments as outlined. For confocal microscopy analysis, the transfected CHO Flp-In cells were added to warm Ham’s F12 Nutrient mix medium to achieve a final concentration of 0.6 × 10^6^ cells/mL in 2 mL. Where ‘exponential’, ‘stationary’, ‘arrested’ and ‘released’ cultures (described in Table 1) were transfected via electroporation, cell cultures were centrifuged at 200 *g* for 5 min and supernatant was discarded. Cell pellets were then resuspended in the relevant culture medium to achieve the aforementioned viable cell density before transfecting in the cuvette. The contents of the cuvette was then transferred to the appropriate medium for maintenance.

**TABLE 1:** Summary of culture manipulation strategies used before and after transfection procedures.

Culture condition	Manipulation strategy prior to transfection	Manipulation strategy post-transfection
Exponential	Cells seeded at 0.2 × 10^6^ viable cells/mL 2 days prior to transfection	None
Stationary	Cells seeded at 0.2 × 10^6^ viable cells/mL 5 days prior to transfection	None
Arrested	Cells seeded at 0.2 × 10^6^ viable cells/mL 2 days prior to transfection- Cells cultured with 9 µM RO3306 18 h before transfection	9 µM RO3306 added to culture medium post transfection
Released	Cells seeded at 0.2 × 10^6^ viable cells/mL 2 days prior to transfection- Cells cultured with 9 µM RO3306 18 h before transfection but cultured in RO3306 free medium for 2 h before transfection	None

#### 2.2.5 Lipofectamine 2000 transfection

Invitrogen’s Flp-In CHO cells were transfected using lipofectamine 2000 (Invitrogen). 2 mL of cells were seeded at 0.6 × 10^6^ cells/mL the day before transfection in a 35 × 10 mm confocal dish (723–100350, Gentaur). 18 h later 2.5 µg of plasmid DNA was diluted in Optimem medium to a total volume of 150 μL and 15 µL lipofectamine 2000 was diluted in 135 µL Optimem medium. The two mixtures were incubated at room temperature separately for 5 min before combining these and incubating the transfection mixture for a further 20 min. The incubated transfection mix was then added directly to cells previously prepared in the confocal dish.

#### 2.2.6 Polyethylenimine (PEI) transfection

40 kDa PEI Max (polysciences) was used to transfect Gibco CHO-S cells and HEK FreeStyle™ 293-F cells in suspension. Cells were first seeded at 0.5 × 10^6^ cells/mL in 125 mL Erlenmeyer flasks in a total culture volume of 20 mL. 500 μg plasmid DNA was diluted in CD-CHO medium (CHO-S) or FreeStyle™ 293 Expression medium (HEK FreeStyle™ 293-F cells) to achieve a total volume of 1.5 mL. An equal volume of PEI Max, which had previously been diluted to a working concentration of 1 mg/mL as per the manufacturer’s instructions, was added to the DNA and incubated for 20 min at room temperature before adding 120 µL to the previously prepared 20 mL culture. This process ensured that 20 µg plasmid DNA was transfected at a DNA:PEI ratio of 1:3 (N/P ratio is 23.2:1). Where “exponential”, “stationary”, “arrested” and “released” cultures (described in Table 1) were transfected via PEI, cell cultures were centrifuged at 200 *g* for 5 min and supernatant was discarded. Cell pellets were then resuspended in the relevant culture medium to achieve the aforementioned viable cell density. At this point the DNA:PEI mix was added to the cell cultures.

### 2.3 Analytical techniques

#### 2.3.1 Confocal microscopy

A Zeiss LSM 880/Elyra/Axio Observer Z1 confocal microscope instrument was used to capture images and videos of cells post-transfection. After transfection (Section 3.2), a confocal dish containing transfected cells was placed on the thermal regulated stage maintained at an environment of 37°C and 5% CO_2_. Fluorescence from eGFP expression was observed by exciting the sample with the 488 nm laser (0.1% intensity) and collecting on the confocal detector. The photomultiplier tube (PMT) light microscopy images were collected concurrently with the eGFP channel. Fluorescence linked to the rhodamine label on the plasmid DNA (see Section 2.1) was collected by exciting the sample with a 561 nm laser (0.1% intensity) and emission was collected on airyscan detectors. Images were collected at ×63 magnification using the “tile” function (4 × 4) to increase the field of view area whilst maintaining higher resolutions. The tiled image was captured every 4 min and 36 s for the duration of the experiment and Zeiss Zen blue software was used to analyse images following image collection where airyscan and stitched processing was applied to images and videos as appropriate.

#### 2.3.2 Flow cytometry

**FIGURE 5:**
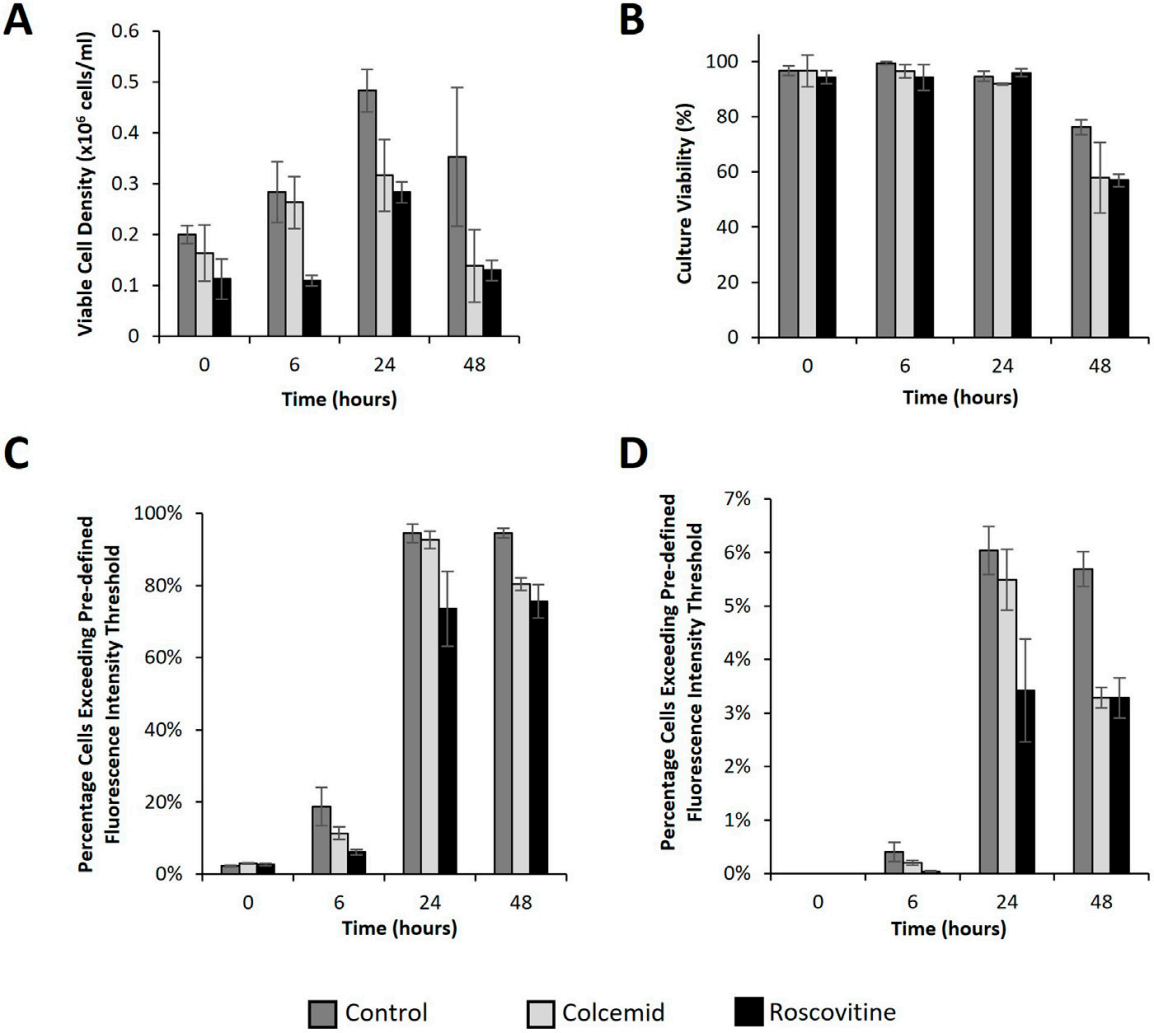
Flow cytometry analysis of CHO cells transfected post treatment with cell cycle arresting drugs. Analysis of CHO Flp-In cells at 0, 6, 24 and 48 h post transfection with a plasmid bearing the eGFP gene using lipofectamine 2000. Cells were cultured with either colcemid (which arrests cells in metaphase), roscovitine (which arrests cells in G1 and G2 phase) or nothing (control) overnight prior to transfection. A ViCell (BD biosciences) was used to monitor viable cell number **(A)** and viability **(B)** and samples were simultaneously harvested to analyse cell fluorescence using flow cytometry. Figure **(C)** shows the percentage of cells fluorescing over a pre-defined threshold applied to determine what percentage of the population expressed eGFP to any level. Figure **(D)** shows the percentage of cells exceeding a pre-defined fluorescence intensity threshold at 100 fold higher intensity than the threshold applied in figure **(C)**. This indicates the “highest” expressors within the population. (n = 3).

**FIGURE 6:**
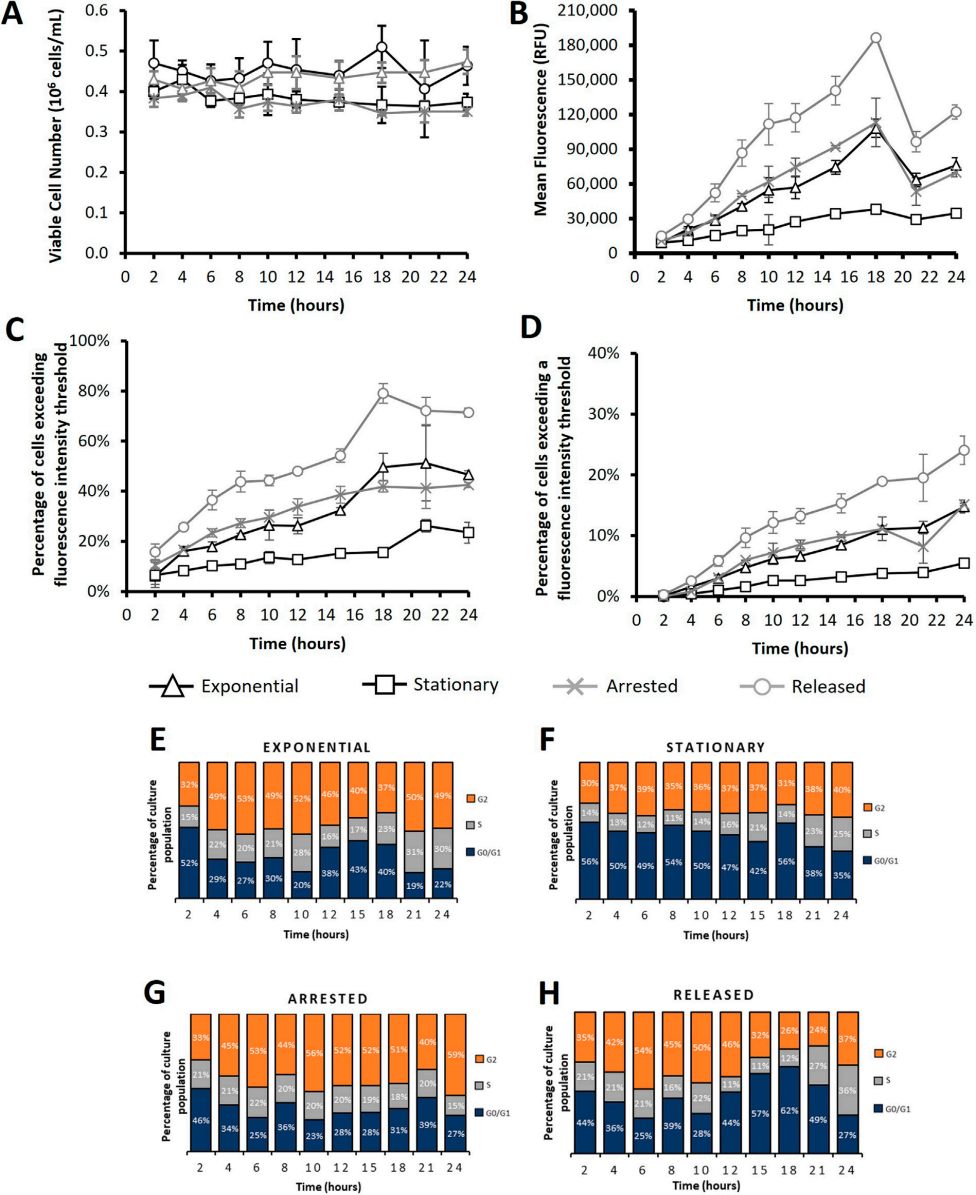
Flow cytometry analysis of CHO cells transfected using electroporation after subjecting cultures to different cell cycle arresting strategies. “*Exponential*” refers to cells which were transfected 2 days after seeding so that culture was in exponential growth phase. “*Stationary*” refers to cells which were transfected at 5 days of culture so that cultures were in stationary phase. “*Arrested*” refers to “Exponential” cells which were treated with R0-3306 (a G2 cell cycle arrest drug) which was sustained throughout the time course post transfection. “*Released*” refers to cultures which were treated with RO-3306 overnight where the drug was removed 2 hours prior to transfection and not reinstated post transfection. Viable cell number **(A)**, mean fluorescence **(B)**, percentage of cells exceeding a low threshold (considered expressing cells) **(C)** and percentage of cells fluorescing over a fluorescence intensity threshold 100X greater than the threshold used in figure **(C)** (considered to be high expressers) **(D)**. Figures **(E–H)** show cell cycle analysis of different cultures over a 24 h period where all plotted results are within 10% standard deviation of the mean. Statistical analysis is summarised in Supplementary Figure 3 (n = 3).

**FIGURE 7:**
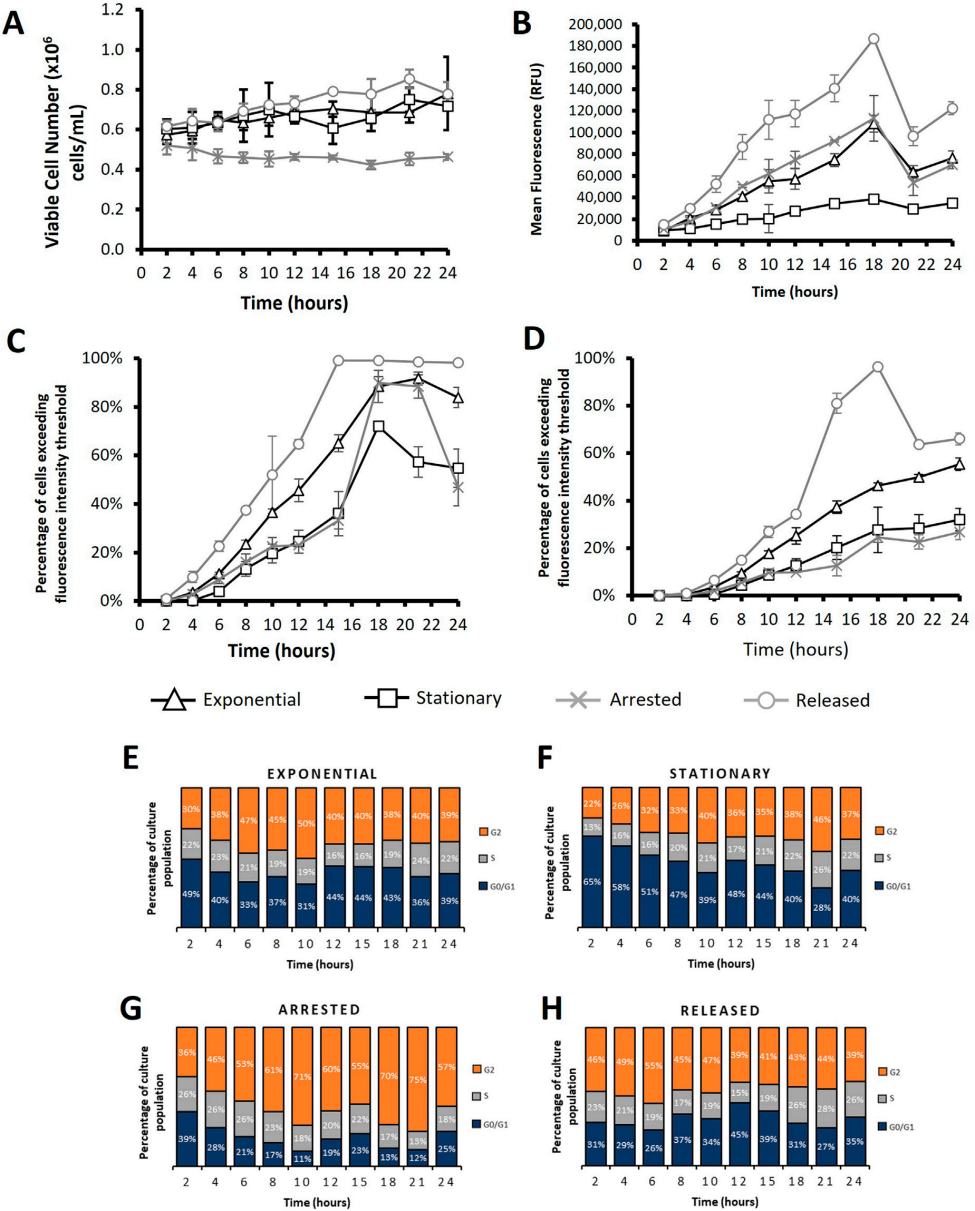
Flow cytometry analysis of CHO-S cells transfected using PEI after subjecting cultures to different cell cycle arresting strategies. “*Exponential*” refers to cells which were transfected 2 days after seeding so that culture was in exponential growth phase. “*Stationary*” refers to cells which were transfected at 5 days of culture so that cultures were in stationary phase. “*Arrested*” refers to “Exponential” cells which were treated with R0-3306 (a G2 cell cycle arrest drug) which was sustained throughout the time course post transfection. “*Released*” refers to cultures which were treated with RO-3306 overnight where the drug was removed 2 hours prior to transfection and not reinstated post transfection. Viable cell number **(A)**, mean fluorescence **(B)**, percentage of cells exceeding a low threshold (considered expressing cells) **(C)** and percentage of cells fluorescing over a fluorescence intensity threshold 100X greater than the threshold used in figure **(C)** (considered to be high expressers) **(D)**. Figures **(E–H)** show cell cycle analysis of different cultures over a 24 h period where all plotted results are within 10% standard deviation of the mean. Statistical analysis is summarised in Supplementary Figure 3 (n = 3).

**FIGURE 8:**
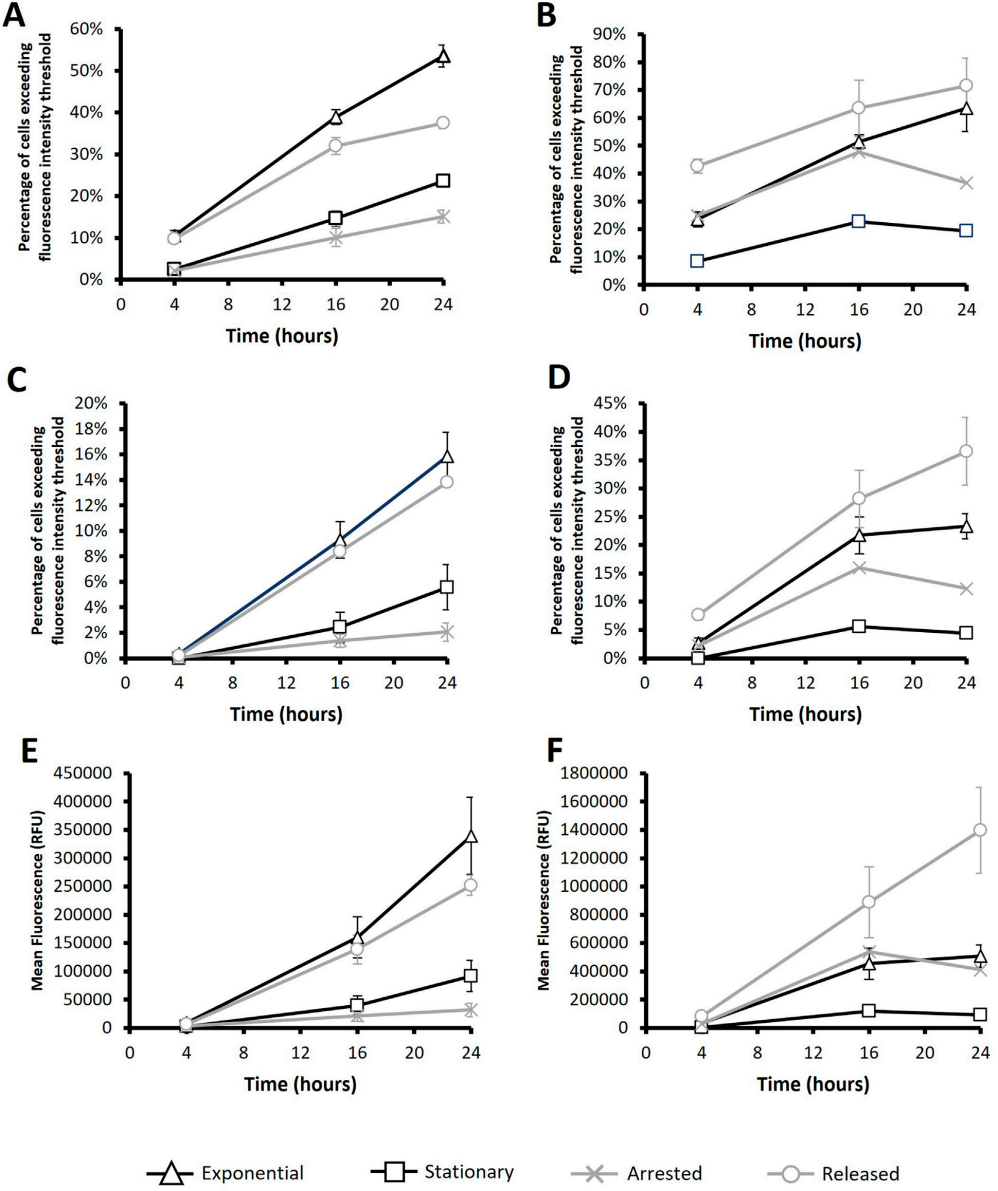
Flow cytometry analysis of HEK293-F cells post transfection with PEI or Electroporation. PEI **(A, C and E)** or Electroporation **(B, D and F)** methods were used to transfect a plasmid encoding the *eGFP* gene after subjecting cultures to different conditions. “*Exponential*” refers to cells which were transfected 2 days after seeding so that culture was in exponential growth phase. “*Stationary*” refers to cells which were transfected at 5 days of culture so that cultures were in stationary phase. “*Arrested*” refers to “Exponential” cells which were treated with R0-3306 (a G2 cell cycle arrest drug) which was sustained throughout the timecourse post transfection. “*Released*” refers to cultures which were treated with RO-3306 overnight where the drug was removed 2 hours prior to transfection and not reinstated post transfection. Flow cytometry analysis was carried out at 4, 16 and 24 h post transfection. The percentage of cells exceeding a low threshold (considered expressing cells) **(A, B)** and percentage of cells fluorescing over a fluorescence intensity threshold 100X greater than the threshold used in figures A and B (considered to be high expressers) **(C, D)**. Statistical analysis is summarised in Supplementary Figure 3 n = 3.

Cells were analysed using flow cytometry following transfection with the eGFP encoding pcDNA5 FRT plasmid. Where specified, cells were fixed prior to analysis by centrifuging 250 µL of cells at 200 x g for 5 min, removing the supernatant and then adding 250 µL 4% paraformaldehyde and incubating at room temperature for 15 min. Subsequently, samples were centrifuged again and supernatant was removed before washing with 500 µL PBS. Samples were then stored at 4°C and analysed within 7 days of collection. Propidium iodide (PI) staining was then carried out using FxCycle™ PI/RNase Staining Solution (F10797, Invitrogen™). Previously fixed pellet samples were resuspended in 100 µL of FxCycle solution and left to incubate for 15 min before analysing the sample on either a FACSJazz™ (BD Biosciences) (Figure 5) or BD Accuri™ C6 plus (BD Biosciences) (Figures 6–8; Supplementary Figure 2). 10,000 events were collected for each sample to ensure that recorded data was reflective of the whole population. All fluorophores used were excited using 488 nm lasers, fluorescence resulting from eGFP expression post-transfection was collected using the FL1 detectors which utilise 530/30 and 533/30 filters for the FACSJazz and Accuri instruments respectively. Additionally, fluorescence from FxCycle™ PI was collected using FL3 detector which utilises the >670 nm filter (whilst the FL2 detector can be used to detect PI stains, the FL3 detector was chosen to exclude any fluorescence from eGFP which may have bled through). Regardless of which flow cytometry instrument was used, gates were generated to exclude fragmented cells and debris as well as doublets. Untreated host cells were used to determine thresholds which enabled identification of non-expressing and expressing cells within the population. The threshold with the lowest fluorescence intensity on the FL1 detector was used to highlight eGFP expressing cells from non-expressing cells and a second threshold was introduced to identify the percentage of cells exceeding a higher fluorescence intensity threshold (10 fold higher than the first threshold). Fluorescence profiles from non-expressing cells were used to gate and analyse the proportion of cells in different stages of the cell cycle.

#### 2.3.3 Western blot analysis

Supernatant samples were harvested by centrifuging 200 µL cultured cells at 200 x g for 5 min 20 μL sample was mixed with 5 µL non-reducing Laemmli buffer (5X) and 20 µL of each sample was loaded on 10% SDS-PA gels and resolved using the Hoefer™ Mighty Small™ II mini vertical electrophoresis system. Proteins which had been resolved in this manner were then transferred to nitrocellulose membranes using Hoefer™ TE22 mini tank blotting unit as per the manufacturers instructions. Nitrocellulose membranes were then blocked for 30 min in a 5% w/v bovine serum albumin (BSA) made up in 0.1% Tween tris-buffered saline (TBS). An anti-γ chain primary antibody (Sigma I976) diluted to 1:10,000 in 5% BSA w/v 0.1% Tween TBS buffer was then exposed to the nitrocellulose membrane and left overnight at 4°C on a shaking platform. Subsequently, the membrane was exposed to a rabbit secondary antibody-peroxidase conjugate (Sigma A4416) 1:1000 in 5% w/v skimmed milk powder 0.1% Tween TBS buffer for 2 h. The relevant polypeptides were then detected via chemiluminescence using Hyperfilm ECL reagents (*GE Healthcare*). Quantitative densitometry was analysed using *ImageJ* software.

## 3 Results

### 3.1 Nuclear import is mediated through events of the cell cycle and is a potential bottleneck to delivery of DNA for recombinant protein production

Live fluorescent confocal microscopy was used to monitor CHO cells post-transfection with plasmid DNA in order to observe the relationship between events of the cell cycle and delivery of DNA into the nucleus. To achieve this, pcDNA5-FRT-eGFP plasmid DNA was first tagged with rhodamine as described in Section 2.1. Flp-In CHO cells adhered to the bottom of a confocal dish were transfected using lipofectamine 2000 where the transfection mix was added directly to the confocal dish mounted to the stage of the microscope. The first image was recorded directly after the addition of the transfection mix and cells were monitored continuously for 9 h post-transfection. Multimedia component 1 shows a time lapse video of a region of interest captured where key events of the transfection process have been highlighted. Figure 2 shows stationary snapshots from the resulting video where one of many transfection events has been focussed on. The video and images used illustrate the key events involved in transfection and, in particular, entry of the tagged plasmid DNA into the nucleus.

The collected data enabled visualisation of key events which led to expression of eGFP which is observable in the final row of images. Figure 2 exemplifies the events observed throughout the experiment and has been used to showcase the stages which were consistent in all cells which culminated in eGFP expression. Upon transfection (T0) no rhodamine tagged plasmid DNA was detected since the lipofectamine/DNA complex had not yet settled to the bottom of the confocal dish or entered cells adhered to the bottom of the culture vessel. Multimedia component 1 shows a steady increase in the abundance of labelled plasmid DNA over time as the lipofectamine/DNA complex settles and enters cells. It is apparent that large areas of rhodamine fluorescence are observed in cells which are yet to divide which is consistent with the understanding that many copies of the plasmid DNA are initially complexed with the lipofectamine 2000 transfection reagent and is shown in the “pre-division” set of images recorded at 120 min.

Initiation of cell division is punctuated by the circularisation of the cell which detaches from the confocal dish surface (“early division”). Simultaneously the rhodamine tagged plasmid DNA disperses throughout the cell and is then observed as small punctate spots suggesting that the DNA has been released from its complex with lipofectamine. The “mid division” images show that DNA is present in the two daughter cells beginning to separate and this remains consistent “post division”. In this example, the entire division event spans a 48 min time period which includes the detaching from the dish surface and adhering of the two daughter cells. This was a typical timeframe for these events to occur during this experiment. It is noted that frequent and prolonged exposure to microscope lasers can impact events such as cell division (Icha et al., 2017).

Fluorescence as a result of eGFP expression was first observed 70 min after division and consistently occurred in both daughter cells. Tagged plasmid DNA was still observed in small punctate spots in recently divided cells but larger complexes were also observed. Curiously, the majority of fluorescence associated with plasmid DNA was still observed at the nuclear periphery but a small amount was observed inside the nucleus (in punctate spots) in contrast to non-divided cells which did not harbour tagged DNA in the nucleus and did not show observable eGFP expression. In addition, Supplementary Figure 1 shows the same analysis of CHO Flp-In cells transfected using PEI mediated transfection. The cellular events highlighted in this figure align with those observed in lipofectamine transfected cells (Figure 2) suggesting that the mechanisms behind DNA delivery are broadly similar. This result was predictable given the comparability of the methods; forming polyplexes and entering cells via endocytosis.

### 3.2 Endosomal escape of exogenous DNA during PEI and lipofectamine transfection occurs early in cell division and is crucial for subsequent import of DNA to the nucleus

Confocal microscopy analysis highlighted differences between mechanisms underpinning successful transfection events when using different transfection techniques. Electroporation, PEI and lipofectamine 2000 mediated approaches were all used to deliver rhodamine labelled pcDNA5-FRT-eGFP plasmid DNA into CHO Flp-In cells and Figure 3 shows still images taken from live confocal microscopy data used to track the transfected cells. The images presented track cells which resulted in successful transgene expression as evidenced by green fluorescence (shown in the final row of images) as a result of eGFP expression brought about by successful delivery of plasmid DNA into the nucleus. Apart from the final row, all other images show only the PMT and rhodamine (red) channels in order to track exogenous DNA during transfection. Cells shown in Figure 3 have been chosen as they exemplify the processes frequently observed for each transfection method.

Regardless of the transfection method used, no pDNA was observable in the nucleus of the transfected cells in the pre division images and was distinctly perinuclear. Moreover, pDNA in the electroporated cell showed a more diffuse and punctate pattern in comparison to both PEI and lipofectamine images which showed larger and more intense rhodamine stains due to the formation of DNA polyplexes/lipoplexes during both techniques. These polyplexes/lipoplexes remained intact at the onset of cell division as observed in the “early division” PEI and lipofectamine images but showed dispersal shortly afterwards in “early division 2” images. This phenomenon consistently occurred imminently after initiation of cell division in all recorded events and these findings suggest that endosomal escape and release of exogenous DNA from polyplexes/lipoplexes is governed by early cellular events of mitosis. Whilst some polyplexes or lipoplexes remained intact throughout the time course, much of the DNA was dispersed ubiquitously in the cell (in the absence of an observable nucleus) shown by punctate red spots. This pattern was consistent in all subsequent images and showed that pDNA was present in small quantities in the nucleus upon reformation in both daughter cells. When using PEI and lipofectamine mediated transfection, all cells which resulted in eGFP expression after a division event, showed both a DNA dispersal event and evidence of successful migration of pDNA into the nucleus of both daughter cells.

### 3.3 The majority of successful transfection occurrences are linked to cell division events but the exact proportion is dependent on transfection method

To quantify the number of cells which expressed eGFP following a cell division event, a population of Flp-In CHO cells transfected using either lipofectamine 2000 or electroporation were monitored using live confocal microscopy over a 12 h period post-transfection. Figure 4A shows these populations at 0, 4, 8 and 12 h where all channels have been merged highlighting the rhodamine tagged pDNA in red and eGFP expression in green overlayed on the PMT image of the cells. It is noted that cells are required to be in suspension for electroporation using the GenePulser instrument and cuvettes used. Electroporated cells were transferred directly to a confocal dish immediately after pulsing but were not adhered to the surface. This is in contrast to lipofectamine which could be transfected in the confocal dish already mounted on the microscope stage. This explains the observable difference in cellular morphology at earlier timepoints of 0 and 4 h where electroporated cells are more rounded and clearly unattached to the culture dish surface. Electroporated cells adhere and have a more similar morphology at later time points (8 and 12 h).

A clear difference between electroporated cells and those transfected via lipofectamine was the pattern of pDNA observed (red fluorescence). Electroporated cells generally showed small punctate spots of pDNA which had a less intense fluorescence profile than pDNA observed in cells which had undergone lipofectamine transfection which showed larger and more abundant areas of red fluorescence. This is consistent with the theory that multiple copies of pDNA are complexed with the lipofectamine and enter the cells as a complex. Furthermore, the red fluorescence became increasingly more intense over time in the lipofectamine transfected cells whereas the intensity and frequency of electroporated cells remained consistent across all timepoints. These observations are in line with the mechanisms of action integral to the differing methods of transfection. Electroporation delivers DNA with a single pulse and no additional pDNA is generally delivered after this point. Conversely, lipid-mediated transfection such as lipofectamine relies on cellular endocytosis of assembled complexes, delivering many copies of pDNA in a single complex. It is also relevant that pDNA is observed in almost all cells of both populations but not all cells show observable expression of eGFP within the timeframe monitored.

Supplementary Video S1 shows time lapsed videos of the monitored populations which were transfected using either lipofectamine or electroporation from which still images were taken and summarised in Figure 4A. The videos were analysed to assess the relationship between cell cycle and expression events in real time in the case of electroporated and lipofectamine transfected cells (Figures 4B, C). The cell count shows the cumulative number of cells expressing eGFP whilst the time shows the point at which each expression event was first evident as determined by the microscope image in which the specific cell showed the first indication of increased fluorescence compared to non-expressing cells in the population. It is noteworthy that the electroporated cells show a higher green fluorescence intensity than lipofectamine transfected cells by the end of the time course and eGFP expression events were clearly evident earlier in the electroporated population. Moreover, the expression events observed and recorded were categorised as either ‘related to division’ which refers to expressing cells which underwent division prior to expression or ‘unrelated to division’ where no division event was observed but eGFP expression was still detected. Expression events which were unrelated to division were more prominent in electroporated cells although a single event was observed in lipofectamine transfected cells. Furthermore, these types of events were more frequent early in culture and made up 50% or more of the expression events up until the 4 h timepoint. It is noted that whilst care was taken to immediately transfer cells to the confocal dish post-transfection, and this transfer lasted no more than 2 min, it is possible that some cells in the electroporated population may have divided between pulsing and capturing the first image. Considering the timeframe required for the cells to divide, it is unlikely that this was the case for all of these cells, if any. Nonetheless, the proportion of cells expressing eGFP after a division event was greater than those that expressed in the absence of a preceding division event and this was the case for both monitored populations regardless of the transfection method employed. These data show that cell division events play a key role in determining transfection efficiencies and the evidence presented here suggests that, since the overwhelming majority of cells contain pDNA post-transfection, import of exogenous DNA into the nucleus where it can be transcribed in the early steps of expression is a key limitation.

### 3.4 Prolonged arrest of cell cycle progression in either G1/G2 or metaphase reduces transfection efficiency

To validate the theory that the cell cycle plays a central role in transfection efficiency, experimentation was carried out that focussed on arresting cells in specific phases of the cell cycle during the lipofectamine 2000 transfection protocol. Roscovitine was employed to arrest cells in both G1 and G2; phases where the nuclear membrane is present and intact. Alternatively, colcemid was used to arrest cells in M-phase and specifically metaphase where the nuclear membrane will have been degraded during previous events of mitosis and will be absent in the arrested cells. Arresting cells in these phases of the cell cycle facilitates evaluation of the nuclear membrane as a bottleneck of transfection and therefore expression of a gene of interest. Adherent CHO Flp-In cells were used and a control population, where no cell cycle inhibitors were added, was also transfected. Appropriate cell cycle inhibitors were added the day prior to transfection and were present in the media throughout the experiment.

Figure 5 shows growth (5A), culture viability (5B) and flow cytometry (5C and D) analysis from the transfected cells over a 48 h period. All cultures remained over 95% viable for 24 h but showed a significant drop in viability at the 48 h timepoint likely due to long exposure to the lipofectamine 2000 transfection reagent in combination with a prolonged exposure to cell cycle inhibitor compounds. This would explain why colcemid and roscovitine treated cells had a lower culture viability than the control at the 48 h timepoint. Inhibitor treated cells also had lower viable cell concentration than the control at all measured timepoints which was expected due to the action of the inhibitors in arresting growth. Progression of the cell cycle has been shown to be species dependent and CHO cells have a propensity to progress slowly even in the presence of cell cycle inhibitors (Kung et al., 1990). Despite this, cell proliferation was markedly slower in treated cultures. It was noted that the difference in viable cell concentration between the control and colcemid treated cells was not significant at 0 and 6 h timepoints whilst the roscovitine treated cells were markedly lower. By 24 and 48 h post-transfection, the viable cell concentrations were lower than the control in the inhibitor treated cells compared to the untreated control.

Interestingly, flow cytometry analysis identified differences in the percentage of cells exceeding particular fluorescence intensity thresholds. Figure 5C shows the percentage of cells fluorescing over the base fluorescence intensity as determined using non-transfected and untreated cells. These events are considered to be all of the expressing cells of the population and give an indication of transfection efficiency of a population. Control samples showed the highest percentage of cells exceeding this fluorescence intensity threshold at 6, 24 and 48 h whilst roscovitine consistently showed the lowest values across the same set of timepoints. Importantly, the colcemid treated cells showed a lower percentage of fluorescing cells after 6 h but a similar percentage after 24 h as the control but was always higher than the roscovitine treated cultures. Since the colcemid treated cells were arrested in metaphase when the nuclear membrane is compromised this gives an indication that delivery across the nuclear membrane is a bottleneck to attaining the highest possible transfection efficiencies. Figure 5D shows the percentage of cells fluorescing to an intensity exceeding pre-determined fluorescence threshold which is 100 fold higher than the threshold applied in the analysis in Figure 5C. These cells are considered to be “high expressors” within the population. Whilst the percentage of cells exceeding this threshold are much lower, the pattern of high expressors is similar to the pattern observed in the analysis indicating overall efficiency. These data support the hypothesis that transfection of pDNA into the nucleus at high efficiency are related to events of the cell cycle. Furthermore, arrested cells where the nuclear membrane is absent show better transfection efficiencies (as determined by eGFP expression) than those arrested where the membrane should be intact, again suggesting that delivery across the nuclear membrane is a bottleneck in the transfection process.

### 3.5 Manipulation of the cell cycle to synchronise cell division events enhances transfection efficiencies and antibody titres

The data presented in Sections 3.3 indicates that transfection efficiency, and particularly nuclear import, are greatly enhanced by the events of metaphase where the nuclear membrane is degraded, adding weight to the theory that exogenous DNA enters the nucleus predominantly via passive import. With this in mind, a strategy was designed where cells were synchronised in G2 phase of the cell cycle and released prior to transfection to evaluate whether an increased frequency of division events shortly preceding transfection could improve the rate of nuclear import and, in turn, transfection efficiency.

Figures 6, 7 and Supplementary Figure 2 show analysis of CHO-S Gibco cells following either electroporation or PEI Max transfection with pcDNA5 FRT-eGFP pDNA where different culture conditions were employed to manipulate the cell cycle and achieve different cell cycle patterns. These culture conditions are summarised in Table 1.

Transfections were carried out using either electroporation (Figure 6; Supplementary Figure 2) or PEI Max (Figure 7) and samples were acquired for cell counts and culture viability as well as flow cytometry analysis every 2–3 h post-transfection for a 24 h period. Propidium iodide (PI) cell staining was also employed to monitor the cell cycle profile of cultures across the 24 h timepoints. This experimentation involved transfection of exponential and stationary cell cultures which were defined by the phase of growth that they were in at the onset of the experiment. Preliminary studies (not shown) showed that cells seeded 2 days prior to transfection were actively proliferating and cells seeded 5 days prior to transfection had reached maximum cell densities and were no longer actively dividing. Figure 6 shows results following electroporation where a 300 V exponential pulse was employed and a single cuvette was transfected per culture flask and cells were manipulated as outlined in Table 1. “Exponential” and “stationary” cultures were seeded two and five days prior to culture respectively whilst “released” and “arrested” cultures were seeded 2 days prior to transfection where RO-3306 was added 18 h before transfection and removed from culture medium 2 hours before transfection for “released” cultures and readded to the “arrested” culture medium post transfection. Viable cell concentrations did not deviate significantly over the 24 h timepoint but both “exponential” and “released” cultures ended at higher viable cell concentrations by the final recorded timepoint of 24 h than “stationary” and “arrested” cultures. Supplementary Figure 2 shows data from a similar experiment where cells were transfected via electroporation using 250V and double the seeded cell density (0.8 × 10^6^/mL). A similar trend was observed where “exponential” and “released” cultures outgrew “stationary” and “arrested” cultures within the monitored time period of 24 h. This phenomenon was logical since “arrested” cultures were intentionally synchronised in G2 phase and not allowed to divide and “stationary” cultures do not divide as rapidly as those in exponential phase. Pre-determined fluorescence intensity thresholds were utilised during flow cytometry analysis to give an indication of the percentage of cells fluorescing, as a result of eGFP expression, over the base intensity level of untransfected cells (Figure 6B). Additionally the percentage of “high expressers” as determined by introducing a threshold at a fluorescence intensity 100 fold greater than the first threshold was also analysed (Figure 6C). Analysis showed that “exponential” cultures consistently had a higher percentage of eGFP expressing cells and high expressers than their “stationary” counterparts providing further evidence that events of cell division are key in achieving high transfection efficiency and nuclear import. Interestingly, the “released” cultures showed the highest percentage of expressing cells throughout culture showing consistently higher values than the “exponential” cultures (Figure 6B) but a more obvious increase was observed when evaluating the high expressers where there was a clear increase in the percentage of cells expressing over the higher threshold from 6 h until the final measured timepoint of 24 h (Figure 5C). Flow cytometry analysis showed that cells manipulated using the ‘released’ process had the highest percentage of cells exceeding both the pre-defined fluorescent thresholds confirming they had the highest number of expressing cells and greatest number of high expressing cells. This also translated into the highest recorded mean fluorescence throughout culture. The increase in the number of cells fluorescing over the first fluorescence in “exponential” cultures was more pronounced with around 25% more of the “exponential” cells in culture expressing eGFP than in the “exponential” culture (whereas this difference was only 1.6% when electroporating with 250 V in Supplementary Figure 2). Interestingly, the “arrested” cells had the second highest mean fluorescence as well as percentages exceeding both fluorescence intensity thresholds until the 15 h measured timepoint, after which the values from the “exponential” samples begin to exceed those of the “arrested”. The “stationary” cultures showed the lowest fluorescence values throughout culture which aligns with the previous experiments.

The manipulated CHO-S cultures were also transfected using PEI as an alternative to electroporation where the same amount of DNA per cell was used. Figure 7 shows data obtained from PEI transfected cells; it is noteworthy that these cells reach higher viable cell concentrations than electroporated cells (Figures 7A, 6A respectively) which is likely due to the milder transfection conditions achieved using the PEI. Electroporation punches holes in the cell membrane which has been shown to affect cell health and, in some instances, culture viability (Sherba et al., 2020). Moreover, PEI transfected cells showed considerably higher mean fluorescence values than their electroporated counterparts and had higher transfection efficiencies in general as shown by flow cytometry analysis (Figures 7C, D). Interestingly, different approaches to manipulate cells showed varying levels of effectiveness depending on the transfection method used. Ultimately the “released” cultures yielded the best results regardless of whether electroporation or PEI was used but, whilst “arrested” cells showed higher mean fluorescence than “exponential” cells when using electroporation, the opposite was true for PEI transfected cultures. “Arrested” cultures consistently had the lowest values for mean fluorescence and transfection efficiencies as determined by the number of cells exceeding a fluorescence intensity threshold. PEI transfected “exponential” cultures generally outperformed “arrested” cultures; a trend which aligns in electroporation based experimentation. These trends align with the data described in Figure 4 which suggests that expression of a transgene is less dependent on events of the cell cycle when electroporating CHO cells than when using lipid or polymer mediated delivery technologies such as lipofectamine or PEI. This is the probable reason that “arrested”, non-dividing cultures perform better, in comparison to the “exponential” cultures, when using electroporation than when using lipid mediated transfection approaches. Overall, data presented in Figure 7 shows that manipulation of cell cycle prior to transfection can be effective in achieving higher transfection efficiencies and, in turn, higher expression levels of a transgene using electroporation or PEI alike.

Following the successful implementation of the described culture manipulation approach in CHO cells, the same methodology was evaluated when transfecting HEK Freestyle™ 293-F cells presented in Figure 8. As before, both PEI (8A, 8C and 8E) and electroporation (8B, 8D and 8F), using 250V protocol, were evaluated using flow cytometry and cultures were harvested for analysis at 4, 16 and 24 h post transfection. Interestingly, electroporated HEK cells reached generally higher mean fluorescence values than their PEI transfected equivalents which contrasts with CHO cell analysis where the reverse was true. It is noteworthy that HEK293 cells have a longer doubling time than CHO-S (33 h and 20 h respectively) this may have a bearing on transfection efficiencies considering that previous results have suggested that chemical methods of delivery to be more dependant on events of the cell cycle than electroporation and is the likely explanation that PEI was less effective as a transfection reagent in HEK293 cells. The percentage of fluorescing cells (8B), percentage of “high expressers” (8D) and mean fluorescence (8F) analysis of electroporated HEK293 cells aligned with that of electroporated CHO-S; “released” cultures consistently produced the highest values across all measured timepoints, “exponential” culture values were consistently second highest followed by “arrested” and then “stationary”. However, it is noteworthy that mean fluorescent values of “exponential” and “arrested” cultures are not significantly different across any of the measured timepoints which is consistent with data attained in CHO-S counterparts. Although “released” culture conditions successfully boosted transfection efficiencies and overall mean fluorescence values for CHO-S cells when transfected via electroporation or PEI as well as electroporated HEK Freestyle™ 293-F cells, this was not the case for PEI mediated pDNA delivery in HEK Freestyle™ 293-F cells. “Exponential” conditions produced the highest average values for percentage of fluorescing cells (8A), percentage of “high expressers” (8C) and mean fluorescence (8E) albeit not significantly different from “released” cultures for the latter two. “Arrested” cultures consistently produced the lowest values across all measured timepoints which was consistent with PEI transfected CHO-S cells. The limitations of enhancing transfections efficiencies using the ‘released’ culture conditions prior to PEI mediated pDNA delivery in HEK293 cultures could be explained by the longer doubling time in comparison to CHO. Once in G2, it may take longer for HEK293 cells to undergo mitosis and return to G1 phase meaning that the time between release from synchronisation and transfection is non-optimal for achieving the maximum number of division events. Furthermore, HEK293 cells were cultured in the presence of RO-3306 for 18 h which is shorter than the doubling time so it is unlikely that all cells are synchronised in G2 upon transfection of the pDNA which could limit its effectiveness.

**FIGURE 9:**
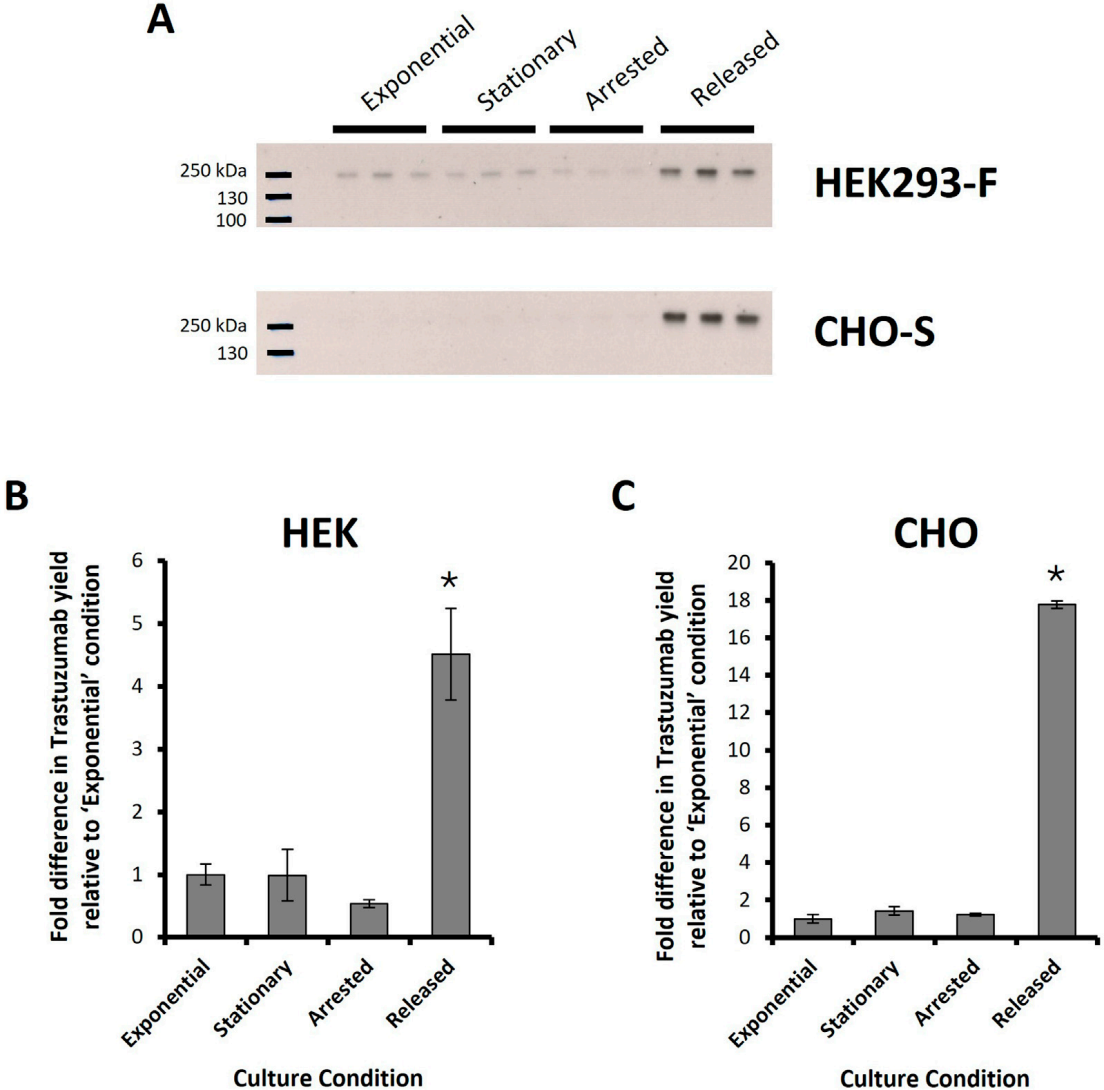
Trastuzumab production in HEK Freestyle™ 293-F and CHO-S cells. PEI-mediated transfections were carried out on HEK293 and CHO cells with a pDNA construct bearing necessary light and heavy chain genes for expression of Trastuzumab. Different culture conditions were used prior to transfection in order to manipulate the cell cycle and are summarised in Table 1. Briefly these conditions are “*Exponential*”*-* cells transfected 2 days after seeding so that culture was in exponential growth phase. “*Stationary*” refers to cells which were transfected at 5 days of culture so that cultures were in stationary phase. “*Arrested*” refers to “Exponential” cells which were treated with R0-3306 (a G2 cell cycle arrest drug) which was sustained throughout the timecourse post transfection. “*Released*” refers to cultures which were treated with RO-3306 overnight where the drug was removed four or 2 hours prior to transfection (for HEK293 and CHO-S cultures respectively) and not reinstated post transfection. Western blot analysis was carried on supernatant samples of transfected cell cultures harvested 24 h post transfection **(A)**. Densitometry analysis was carried out on these western blots and relative band intensities are shown for HEK293-F **(B)** and CHO-S **(C)** derived samples where fold differences are normalised to “Exponential” values. (n = 3; * = p < 0.05 analysed against all other measured samples using ANOVA and *post hoc* Tukey Kramer test).

Finally, the approach of synchronising cells in G2 phase and releasing them in a timely manner in order to maximise cell division events post transfection was evaluated in both CHO-S and HEK293 cells where PEI methods were used to deliver pDNA encoding trastuzumab light and heavy chain genes. Western blot analysis and subsequent densitometry was used to determine the relative production of trastuzumab in supernatant samples harvested 24 h post transfection where the same culture conditions, previously described, were employed (Figure 9). ‘Released’ HEK293 cultures were performed as previously described but RO-3306 was removed 4 hours before transfection (as opposed to 2 hours used for CHO-S cells in this experiment and in all previously described experiments; Figures 6–8) as it was hypothesised that this timing may achieve the benefits from HEK293 cells which was not evident in data presented in Figure 8. The band intensities were plotted relative to “exponential” data which was plotted at 1 (au) and “released” HEK293 cultures showed a 4.5 fold increase in comparison (Figure 9B). Arrested samples showed the lowest trastuzumab abundance, which aligns with previous data, but no significant difference was observed between “exponential” and “stationary” cultures. Figure 9C shows corresponding results in CHO where “exponential”, “stationary” and “arrested” cultures showed similar abundances of trastuzumab, “released” showed a relative increase of almost 18-fold over the ‘exponential’ conditions. This increase aligns with the trends observed in transfection efficiency data (Figure 7) but far exceeds the percentage increase in transfection efficiency. Secreted molecules such as trastuzumab accumulate in the culture medium post synthesis and secretion meaning that increased production early in culture will impact titres observed at later timepoints. This is likely a major factor in the vast increase in trastuzumab production which was not observed in eGFP reporter based experiments as eGFP is a non-secreted protein.

## 4 Discussion

Manipulation of the cell cycle has previously been utilised to enhance recombinant biotherapeutic production in mammalian cells by controlling viable cell concentrations (Xu et al., 2019; Xu et al., 2023) and prolonging apoptosis (Baek et al., 2017; Henry et al., 2020; Xiong et al., 2019). Maximising titres of recombinant molecules can also be dependent on optimisation of transfection techniques in both transient production and stable cell line generation (Gong and Wu, 2023). Furthermore, delivery of exogenous genetic material such as pDNA into mammalian cells for expression of a transgene of interest has previously been shown to be dependent on cell culture phase profiles at the point of DNA transfection initiation and the method of transfection (Brunner et al., 2000; Cervia et al., 2018; Golzio et al., 2002; Grosse et al., 2006; Stroh et al., 2010; Shi et al., 2018). Numerous reports show that overall transfection efficiency is far greater in actively dividing cells than in non-dividing cells (Brunner et al., 2000; Fasbender et al., 1997; Grosjean et al., 2002; Li et al., 2013; Ludtke et al., 2002; Sylvers et al., 2023; Tait et al., 2004). Delivery of exogenous DNA into the cell nucleus is a necessary step for expression of a gene of interest but the cellular mechanisms behind nuclear import of exogenous DNA remains poorly defined and has been identified as a potential bottleneck in production of a recombinant protein (Lam and Dean, 2010). Previous attempts have been made to observe cellular events involved in delivery of exogenous DNA into the nucleus via lipid mediated transfection methods and observations align with data presented herein (Greitens et al., 2024; Haraguchi et al., 2022; Haraguchi et al., 2001; Sylvers et al., 2023; Vaughan and Dean, 2006). In this study, the entire process of gene expression has been observed from initial transfection of pDNA to final expression of a eGFP reporter gene. Observations show, in line with previous reports, that delivery of DNA into mammalian cells is predominantly linked to events of the cell cycle and the events involved in cationic lipid mediated transfection technologies can be summarised as (1) endocytosis of DNA into the cytosol, (2) endosomal rupture coinciding with dynamic dispersal of DNA during mitosis, and (3) passive encapsulation of DNA in the nucleus. Expression of a gene of interest is then observed in each of the resulting daughter cells succeeding mitosis.

*Haraguchi et al.* have reported the same set of events which occur during transfection of HeLa cells. The study by these authors highlighted the role of barrier-to-autointegration factor (BAF) which can bind to transfected DNA, inhibiting autophagy, and has properties in nuclear envelope (NE) reformation during telophase of mitosis; gene expression was delayed when BAF was knocked down (Haraguchi et al., 2022). Cell cycle progression is dynamically controlled through a symphony of highly complex and organised gene regulation which, in turn, regulates cellular events including cell growth, replication of genetic material and duplication of organelles, and finally division to form two daughter cells (Kittler et al., 2008). Endosome rupture and dispersal of exogenous DNA has emerged as a key event in the transfection process and has been shown to occur during mitosis. The specific and consistent timing of these events indicate that the rupture may be associated with the onset of differential endogenous gene expression linked with mitosis during cell cycle progression.

In addition to successful transfection incidents which were linked to cell division events, a small proportion of cells successfully expressed eGFP without preceding cell division and this phenomenon has been previously reported by others (Akita et al., 2015; Dean, 1997; Dowty et al., 1995; Munkonge et al., 2009). Successful transfection which is independent of cell cycle events occurs at a higher frequency in an electroporated population than lipofectamine transfected cells. Electroporation methods punch holes in the cell membrane and delivers naked exogenous DNA into the cell (i.e., DNA is not contained in an endosome) and therefore does not require endosomal rupture which is integral to the vast majority of successful transfection events in lipid mediated delivery. This is supported by the difference in DNA patterning seen in electroporated cells versus lipofectamine transfected cells shown in this study. Regardless, efficient transfection in an electroporated population is still dependent on active cell division and this is likely related to the requirement to subsequently traverse the NE which predominantly appears to occur via passive encapsulation upon reformation at telophase of mitosis. It is also noteworthy that delivery of DNA through the NPC during active transport into the nucleus is dependent on the presence of specific sequences (Dean, 1997; Dean et al., 1999) and can also be affected by size of the DNA molecule transfected (Akita et al., 2015).

Cell cycle manipulation has previously been studied to ascertain the effect of transfecting cells when synchronised at different stages of the cell cycle with a consensus that transfection at later stages of the cell cycle (S or G2) leads to optimal efficiency and depends on cell type and transfection method (Brunner et al., 2000; Golzio et al., 2002; Grosjean et al., 2002; Ludtke et al., 2002). Data in the current study aligns with these findings and suggests that the reason for increased efficiency is the synchronisation of mitotic events such that the frequency is highest directly after transfection. This hypothesis was considered and examined through design of a novel transfection protocol which focusses on manipulation of CHO and HEK cells such that they are synchronised at G2 phase of the cell cycle, directly before initiation of mitosis, using RO-3306. The cell cycle inhibitor was then released two or four hours before initiation of transfection (for CHO and HEK cells respectively), the optimal time for reversal of the effect of RO-3306 (as determined by others and confirmed experimentally prior to design of the transfection technique (Schaefer et al., 2022)). This approach was successful in enhancing transfection efficiencies and overall mean fluorescence as a result of eGFP expression regardless of whether electroporation or chemical polymer based transfection techniques were used. The notable exception to this was using PEI mediated transfection on HEK293 cells where releasing the culture from G2 phase and allowing two hours in the absence of RO-3306 did not enhance transfection efficiencies as it did with the electroporated cells and in CHO-S cells. The timing of release and transfection has proved crucial in the success of the described method and culturing cells in the absence of RO-3306 for four hours following its removal did result in enhanced trastuzumab production. It is noteworthy that sustaining arrest of cultures in G2 phase following transfection affected cells differently depending on the transfection method. Electroporated cultures showed relatively higher transfection efficiencies than the untreated “exponential” controls post transfection via electroporation but consistently lower transfection efficiencies in PEI transfected counterparts and this was consistent for both CHO and HEK293 cells. This adds weight to the theory that cells transfected with chemical polymer mediated transfection methods are more dependent on events of the cell cycle. Cultures transfected during exponential phase consistently yielded higher transfection efficiencies than cultures transfected during stationary phase and this was true for electroporation and PEI methods. Stationary cultures generally showed the highest percentage of cells in G1 phase throughout culture. It is likely that the frequency of mitotic events throughout the 24 h experiment was lowest in stationary cells and explains why these cells had lower transfection efficiencies than exponential cultures. The same amount of DNA per cell was used for all transfections presented in Figures 6–8 and it is noticeable that PEI transfection was superior in obtaining higher maximum transfection efficiencies and mean fluorescence values in CHO cells but the opposite is true in HEK293 cells. Rapid degradation of DNA in the cytoplasm can limit transfection efficiencies (Bai et al., 2017) and it is therefore advantageous to promptly deliver DNA into the nucleus once in the cytoplasm to reduce DNA degradation.

Process design and manipulation of both HEK and CHO cultures resulted in an increase in the production of the monoclonal antibody trastuzumab by 4.5 and 18 fold respectively at 24 h respectively. This strategy was intended to increase transfection efficiencies but it should be noted that while these efficiencies play a significant role in the overall production of a recombinant protein, they are not the only contributing factor; for example, transcription, translation and secretory cellular processes are all necessary to synthesise monoclonal antibodies in mammalian hosts. Previous studies have shown that recombinant protein production is maximal in G1/G0 phase (Avello et al., 2022) and it is possible that, whilst transfection efficiency is likely to be the main reason behind improved trastuzumab production, timely progression to G1/G0 may also contribute. Furthermore, the relationship between the abundance of DNA, mRNA and protein is not a linear relationship (Mead et al., 2009) i.e., one copy of DNA can produce several copies of mRNA which can, in turn, produce several proteins which could account for the substantial increase in trastuzumab despite a smaller increase in overall transfection efficiencies observed in experimentation.

Large scale plasmid DNA production at the scale required for market demand remains a challenge for the industry (Ohlson, 2020; Srivastava et al., 2021). The implications of techniques, such as the one presented here, which yield higher transfection efficiencies and transient recombinant protein production when using the same amount of DNA could reduce the requirement to produce DNA in such vast quantities.

Overall, this study has provided new insight into the fundamental biology involved in transfection of CHO and HEK293 cells and how efficient transfection using either electroporation or chemical polymer based transfection methods is linked with active cell division. Successful expression of a gene of interest relies upon delivery of DNA across the plasma membrane into the cytosol and this event occurred in all cells observed throughout experimentation regardless of the transfection method employed. Subsequent delivery into the nucleus proved a relatively more difficult barrier to overcome and delivery of DNA across the nuclear envelope is a potential bottleneck in expression of a gene of interest. These learnings facilitated rational design and demonstration of a transfection approach which centred around cell cycle synchronisation in G2 phase and timely release to increase the frequency of mitotic events occurring directly after transfection. This lead to enhanced transfection efficiencies, a higher percentage of cells expressing the highest levels of eGFP and the highest overall mean fluorescence as a result of transgene expression in cultures transfected using PEI or electroporation methods. These findings can be utilised to inform development of existing and novel techniques and technologies requiring delivery of DNA into mammalian cells.

## Data Availability

The raw data supporting the conclusions of this article will be made available by the authors, without undue reservation.
