# Supplementary figures and images for: Mapping cellular processes that determine delivery of plasmid DNA to the nucleus: application in Chinese hamster ovary and human embryonic kidney cells to enhance protein production
Source: Front Bioeng Biotechnol. 2025 Mar 21;13:1466671. doi: 10.3389/fbioe.2025.1466671 (PMC11969153; doi:10.3389/fbioe.2025.1466671)

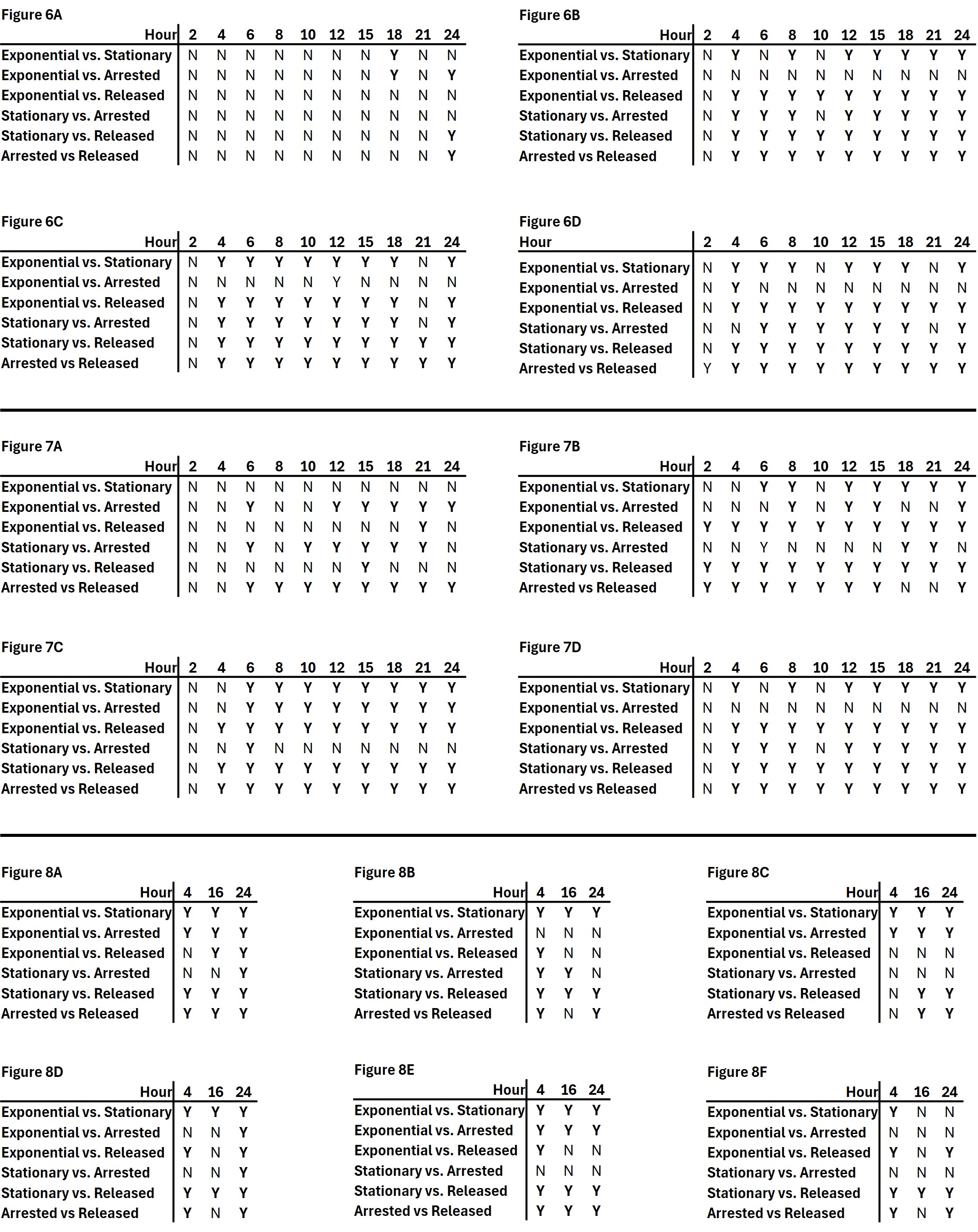

Supplement: Supplementary file 1 [file Image3.jpg]

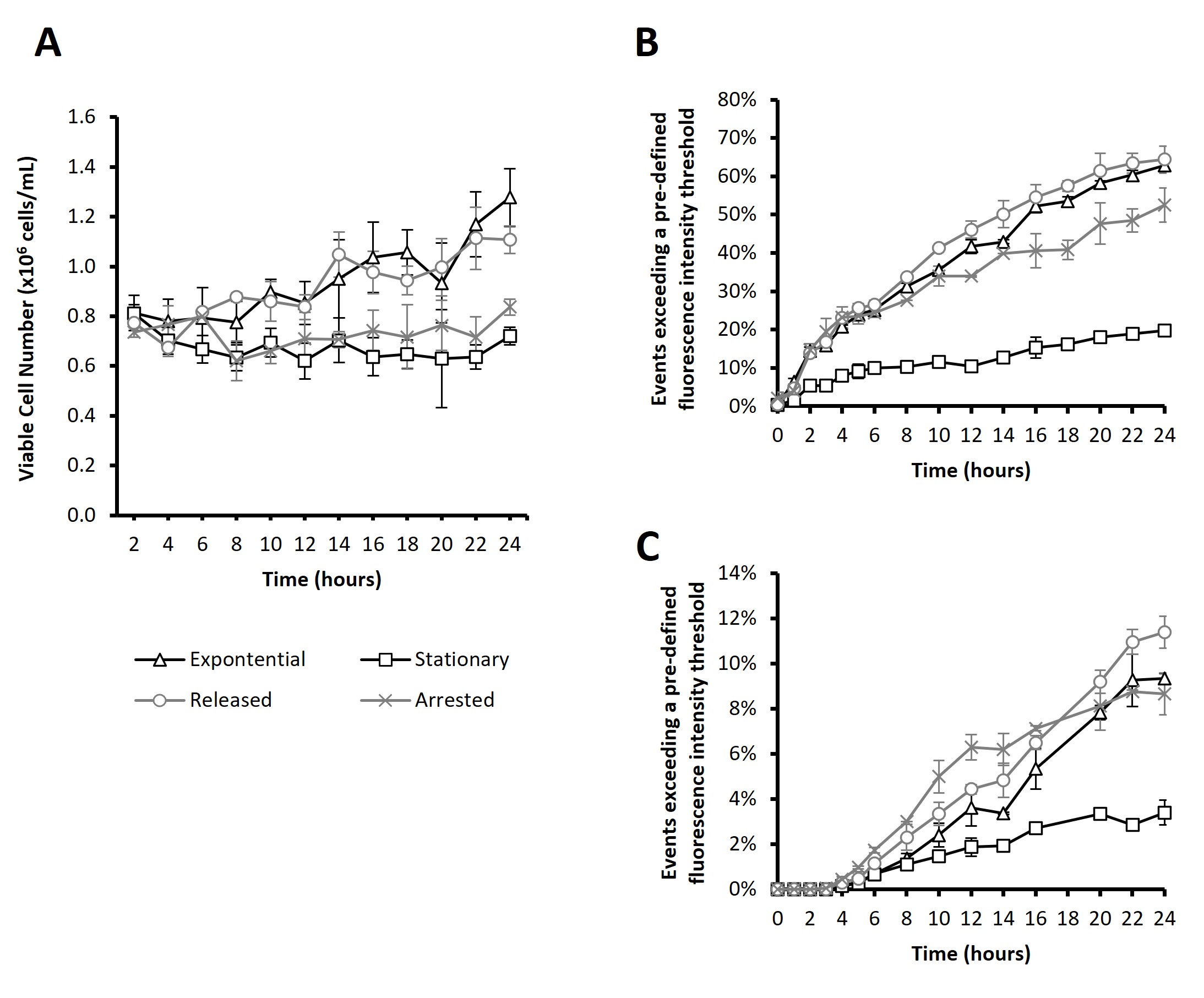

Supplement: Supplementary file 2 [file Image2.jpg]

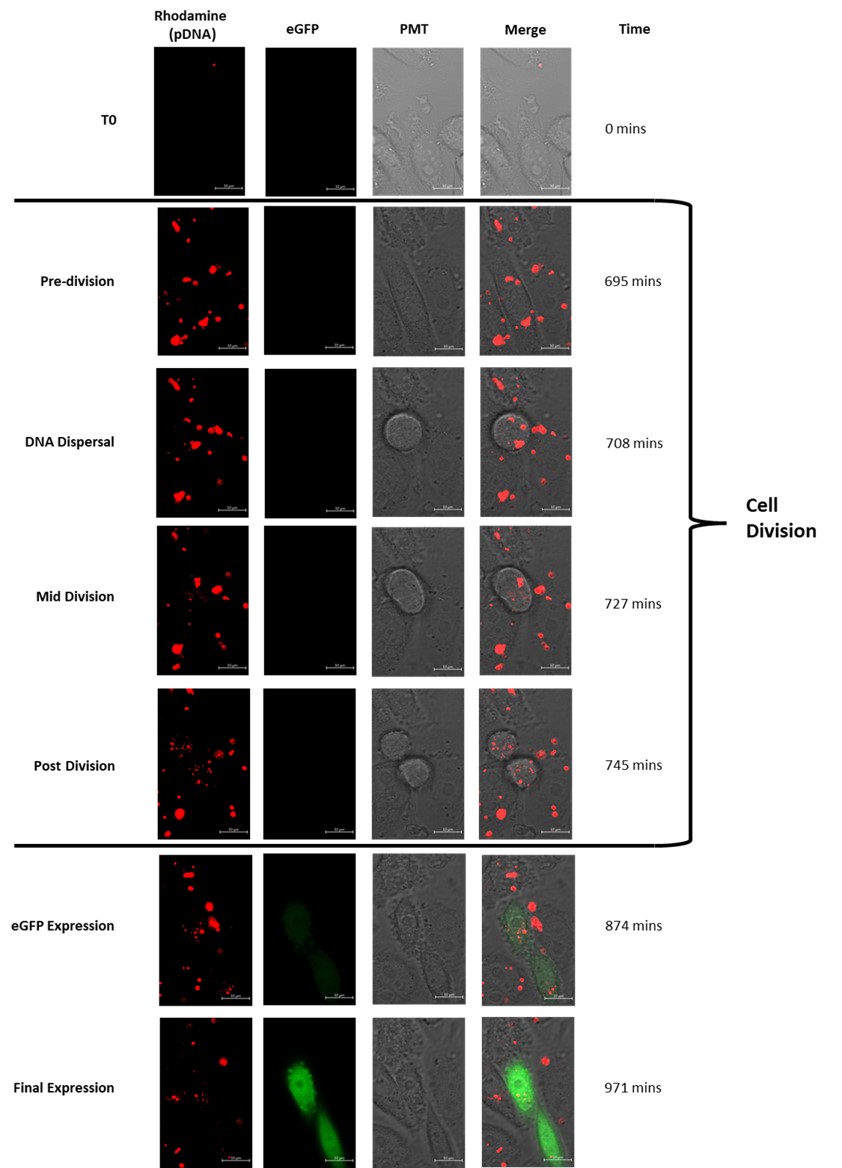

Supplement: Supplementary file 4 [file Image1.jpg]
